# HyRA: A Hybrid Recommendation Algorithm Focused on Smart POI. Ceutí as a Study Scenario

**DOI:** 10.3390/s18030890

**Published:** 2018-03-17

**Authors:** Joanna Alvarado-Uribe, Andrea Gómez-Oliva, Ari Yair Barrera-Animas, Germán Molina, Miguel Gonzalez-Mendoza, María Concepción Parra-Meroño, Antonio J. Jara

**Affiliations:** 1 Computer Science Department, Tecnologico de Monterrey, School of Engineering and Sciences, Carretera Lago de Guadalupe Km. 3.5, Col. Margarita Maza de Juárez, Atizapán de Zaragoza 52926, Estado de Mexico, Mexico; ybarrera@itesm.mx (A.Y.B.-A.); mgonza@itesm.mx (M.G.-M.); 2 HOP Ubiquitous S.L., Calle Luis Buñuel No. 6, 30562 Ceutí, Murcia, Spain; andrea@hopu.eu (A.G.-O.); german@hopu.eu (G.M.); 3 Social Sciences, Law and Business Department, Universidad Católica de Murcia (UCAM),Business Administration, Marketing and Economics, Campus de los Jerónimos, Guadalupe, 30107 Murcia, Spain; agomez14@alu.ucam.edu (A.G.-O.); mcparra@ucam.edu (M.C.P.-M.); 4 Institute of Information Systems, University of Applied Sciences Western Switzerland, ConEx Lab, 3960 Sierre, Switzerland; jara@ieee.org

**Keywords:** recommendation algorithm, point-of-interest, similarity and distance measures, aggregation operator, POI category, geographical influence, tourism

## Abstract

Nowadays, Physical Web together with the increase in the use of mobile devices, Global Positioning System (GPS), and Social Networking Sites (SNS) have caused users to share enriched information on the Web such as their tourist experiences. Therefore, an area that has been significantly improved by using the contextual information provided by these technologies is tourism. In this way, the main goals of this work are to propose and develop an algorithm focused on the recommendation of Smart Point of Interaction (Smart POI) for a specific user according to his/her preferences and the Smart POIs’ context. Hence, a novel Hybrid Recommendation Algorithm (HyRA) is presented by incorporating an aggregation operator into the user-based Collaborative Filtering (CF) algorithm as well as including the Smart POIs’ categories and geographical information. For the experimental phase, two real-world datasets have been collected and preprocessed. In addition, one Smart POIs’ categories dataset was built. As a result, a dataset composed of 16 Smart POIs, another constituted by the explicit preferences of 200 respondents, and the last dataset integrated by 13 Smart POIs’ categories are provided. The experimental results show that the recommendations suggested by HyRA are promising.

## 1. Introduction

Nowadays, Physical Web [1] together with the increase in the use of mobile devices, Global Positioning System (GPS), and Social Networking Sites (SNS) have caused users to share enriched information on the Web such as their tourist experiences [2]. Nevertheless, generally, tourist guide applications are based on information heavily related to the location, disregarding other types of context information, which can be provided by the user. Consequently, all information is available to all users in touch, leading to the issue known as “information overload” [3] as well as problems of inappropriate suggestions [4]. Such facts entail the need to enhance the user’s individual tourist experience according to his/her preferences and context information.

Hence, the main issue to be addressed is recommending a new point-of-interest (POI) where users might be interested based on their personal preferences and contextual information. On the one hand, such a statement matches with the problem of POI recommendation, i.e., the difficulty of suggesting personalized recommendations of places of interest, such as restaurants and movie theaters, for users [2,5,6]. On the other hand, this approach also coincides with one of the benefits of POI recommendation: to help both residents and visitors to explore new and interesting places in a certain area [7].

In accordance with the foregoing, it is noteworthy that the traditional recommendation systems have been widely addressed in these systems, where the user generally provides ratings to the items, such as books, movies, music, among others [2]. However, the POI recommendation systems have just emerged recently [2] as a consequence of the quick development of new location-based technologies. Specifically, this approach will deal with Google’s Physical Web technology [1] integrated into a device called Smart Spot, and the concept of Smart Point of Interaction (Smart POI) defined as a smart point of interaction between users (citizens and visitors) and a Smart Spot [8,9]—a technology and a device that will be used for the first time in a research study related to the formalization of a recommendation algorithm in the tourism sector.

Therefore, the main goals of this work are to propose and develop an algorithm that recommends a Smart POI list for a user according to the user preferences, the Smart POIs’ contextual information such as categories and geographical information, and the characteristics of Smart Spot in conjunction with the definition of Smart POI. In other words, the hypothesis of this proposal is the following: the adaptation of a user-based Collaborative Filtering (CF) algorithm integrating an aggregation operator constituted of different similarity and distance measures allows dealing with the user preferences as well as the characteristics of the technology used in this work, and the incorporation of the Smart POIs’ categories and the geographical influence factor into the modified user-based CF algorithm allows addressing the Smart POIs’ contextual information.

Hence, on the one hand, this proposal could be classified into the user-based CF systems belonging to the memory-based category from the CF systems [7,10], as well as into a content-based system [10]. On the other hand, it could be considered as a POI recommendation system [5,7,10]. It means, firstly, the proposed approach addresses the user’s explicit preferences (ratings) through a user-based CF algorithm that embraces an average aggregation operator integrated by five similarity and distance measures. Secondly, the proposal becomes a content-based system by incorporating the Smart POIs’ categories (also named as tags or topics) into the modified user-based CF algorithm. Thirdly, the proposed approach is considered a POI recommendation system by incorporating the geographical influence factor into the modified user-based CF algorithm. Finally, the proposed approach—called the Hybrid Recommendation Algorithm (HyRA)—encodes the modified user-based CF algorithm along with the Smart POIs’ categories and the geographical influence factor.

To test HyRA, data belonging to both users and Smart POIs are required. Thus, to collect information related to the POIs from Ceutí—a town belonging to the Región de Murcia in Spain—a research study [11] has been considered. In addition, with the aim of generating a dataset with the users’ explicit preferences, two surveys designed in a previous study [11], one in Spanish and the other in English, have been redesigned and disseminated. Lastly, to define the POIs’ categories, the All Categories section from the Yahoo! Answers website [12] as well as the description of the POIs from Ceutí have been reviewed. As a result, three datasets have been produced: one dataset composed of 16 Smart POIs, another constituted by the 16 preferences of 200 respondents, and another consisting of 13 categories.

The experimental results indicate that HyRA recommends a Smart POI list closer to the user preferences than the approaches included in this evaluation. To summarize, the main contributions of this article are six-fold:

1. The use of Google’s Physical Web technology as well as the Smart Spot device in the proposal of a recommendation algorithm in the tourism sector.
2. The incorporation of an average aggregation operator integrated by five similarity and distance measures, validated among a total of nine measures, into a user-based CF algorithm.
3. The HyRA’s proposal and development, encoding the improved user-based CF algorithm along with the Smart POIs’ categories and the geographical influence factor.
4. Two datasets built with real-world information: one dataset composed of 16 Smart POIs (Smart POIs Dataset in Ceutí) and another constituted by the 16 explicit preferences of 200 respondents (User preferences Dataset).
5. One experimental dataset comprised of 13 Smart POIs’ categories (Smart POI’s Categories Dataset).
6. The experimental results show that HyRA provides better recommendations against other approaches.

The rest of the article is organized as follows. Section 2 presents the related work to the traditional and POI recommendation algorithms, as well as the state-of-the-art similarity and distance measures, and POI and Smart POI. Subsequently, Section 3 provides the description and pseudocode of HyRA. Later, Section 4 and Section 5 provide the experimental cases, and the results and discussions about them, respectively. Then, Section 6 addresses the datasets used and generated in this article. Finally, Section 7 gives the conclusions.

## 2. State of the Art

Concepts and approaches concerning the traditional and POI recommendation algorithms will be introduced. In the same way, a brief comparison of related work to the POI recommendation algorithms will be presented. Subsequently, the similarity and distance measures used in this work will be described. Finally, the definitions and characteristics about POI and Smart POI will be explained.

### 2.1. Traditional and POI Recommendation Algorithms

Recommendation systems are based on personalization systems. Amoretti et al. [13] defined a personalization system as a computer-based application that learns the behavior of a person to generate and manage his/her profile. Specifically, when the personalization system can provide suggestions to a user according to his/her profile, then these systems are called as recommendation systems. Such recommendations can be of any type of product or interest, such as places, technology, entertainment, food, and so on. For this reason, the recommendations systems can support other applications and services in adapting to the specific preferences of each user. Netflix, YouTube, and Spotify are a few examples of applications and services that make use of recommendation systems. Consequently, an active and challenging research area is the development of algorithms capable of giving accurate recommendations to users based on their individual preferences.

As previously mentioned, recommendation systems can be used in several contexts. Because the main goals of this research work are to propose and develop a recommendation algorithm to improve the tourist experience of users, this proposal is focused on two types of systems: the traditional recommendation systems and the POI recommendation systems. On the one hand, the traditional recommendation approaches commonly obtain user preferences through ratings that he/she provides to certain items in an application or service, such as books, movies, or music [2,14]. On the other hand, the POI recommendation systems model the users’ visiting preferences in order to recommend POIs that the user never visited before but could be interested in [5,7,15]. Therefore, according to these definitions and the scope of the proposed Smart POI recommendations, this research is mainly focused on the related work to the POI recommendation algorithms. In the following, some approaches about POI recommendation algorithms are briefly described.

**POI Recommendation Approaches for LBSN.** One of the fields of application of the POI recommendation algorithms is the suggestion of POIs for LBSN. Some approaches will be introduced according to the year of publication below. Firstly, Ye et al. [10] proposed a unified POI recommendation framework to provide a POI recommendation service for LBSNs, exploring user preference, social influence, and geographical influence. Later, Zheng et al. [16] proposed the cross-region topic-based collaborative filtering (CRTCF) method based on hidden topics mined from user check-in records with the aim of recommending new POIs to a user in regions where he/she has rarely been before. In the same year, Liu et al. [5] proposed a Geographical-Topical Bayesian Non-negative Matrix Factorization (GT-BNMF) model that allows capturing the geographical influences on user’s check-in behaviors, as well as integrating the POIs’ regional popularity. Similarly, Liu et al. [17] proposed a two-stage category-aware POI recommendation model to suggest a personalized POI based on user’s check-ins, geographical influences, POI categories, and temporal information. Subsequently, Yuan et al. [7] proposed Geographical-Temporal influences Aware Graph (GTAG) to deal with the problem of the time-aware POI recommendation; with GTAG, they intended to model check-in records as well as to exploit both geographical and temporal influences of these records for the time-aware POI recommendation. Afterwards, Liu et al. [6] proposed a general geographical probabilistic factor model (Geo-PFM) framework which can capture the geographical influence on a user’s check-in behavior. In the same year, Zhang and Wang [18] proposed a location and time aware social collaborative retrieval model (LTSCR) for the successive POI recommendation task considering the user’s location, time, and social information simultaneously. Finally, Guo et al. [15] proposed a weighted Bayesian personalized ranking model with visit frequency and distance (WBPR-FD) to give POI recommendations using user’s check-ins and geographical distance.

**POI Recommendation Approaches for Tourism.** Another field of application is the tourism sector. Some approaches will be introduced according to the year of publication below. Firstly, Kang et al. [19] proposed a Personalized POI Recommendation Method for the tourist POI recommendation as well as the POI and user data that can be exploited for this task. Specifically, they used the user’s explicit preferences and POI categories to carry out the tourist recommendations. Later, Ying et al. [20] proposed an Urban POI-Mine (UPOI-Mine) approach to suggest urban POIs based on the users’ check-ins, POI categories and popularity, along with social influence. Subsequently, Meehan et al. [3] proposed a work in progress to deal with problems of inappropriate suggestions arisen information overload and inadequate content filtering by means algorithms implemented in their application in development called as VISIT (Virtual Intelligent System for Informing Tourists), a context-aware tourist app. Finally, Yu et al. [21] proposed a recommender of personalized travel packages with multiple POIs based on crowd-sourced user footprints to help users find interesting locations as well as to generate travel packages consisting of different types of locations and visiting sequences. To carry out the recommendations, crowd-sourced check-in records, ratings, POI categories, geographical influence, and temporal information are considered.

A comparative table summarizing the previously mentioned works is presented to highlight the contributions of this research. The aspects considered are described below:

- Year (Y). It refers to the year of publication of the approach.
- Rating (R). Data that consider the recommendation algorithm to address the user’s explicit preference on POIs.
- Check-in (CI). Data that consider the recommendation algorithm to address the user’s implicit preference on POIs.
- Geographical Influence (GI). Factor that is examined in the POI recommendation approach.
- Social Influence (SI). Factor that is explored in the POI recommendation approach.
- Category (C). Data that consider the recommendation algorithm to address the POI tags, categories, or topics.
- Another context data (ACD). Some other data that the POI recommendation algorithm considers different from the data and factors mentioned in this comparison.
- Information Source (IS). Source that is employed in the collection of the data used to evaluate the POI recommender.
- Similarity and Distance Measures (SDM). Measure that is applied in the POI recommendation algorithm.
- User-based CF with Aggregation Operator (UCF+), where **✗** * indicates that the approach works with the user-based CF algorithm without an aggregation operator. Algorithm that is implemented as a POI recommender using an aggregation operator as a similarity measure.
- Physical Web, Smart Spot, and Smart POI (PSSP). Technology and device that are used to collect the input data of the POI recommendation algorithm.
- Scope (S). Field of application of the approach.

In summary, according to Table 1, it is concluded that only one approach [19] in addition to HyRA addresses ratings, nine of them address check-ins [5,6,7,10,15,16,17,18,20]; and one addresses both ratings and check-ins [21]. Geographical influence is the factor most used than social influence with eight [5,6,7,10,15,17,18,21] (in addition to HyRA) and four [10,16,18,20] works, respectively. The POI categories—six approaches [5,16,17,19,20,21] (in addition to HyRA)—are also more explored than social influence. Other characteristics that have been analyzed are temporal influence by four approaches [7,17,18,21] and POI popularity by one approach [20]. Most approaches used LBSN to collect data for the recommendation algorithm, only two approaches [3,19] and HyRA used other information sources. Cosine similarity is the most common measure in the recommendation approaches [10,16,18,19,20,21], Euclidean distance is used in [20], and Pearson correlation, Euclidean distance, Cosine similarity, Manhattan distance, and Chebyshev distance are used by HyRA. The user-based CF algorithm is employed in [10,19,21] using the Cosine similarity as a similarity measure while HyRA is the only approach that codes a user-based CF with an average aggregation operator as a similarity measure. Similarly, HyRA is the only approach that deals with the Physical Web technology, the Smart Spot device, and the Smart POI concept.

### 2.2. Similarity and Distance Measures

The definitions related to the similarity and distance measures used in this work are described below.

- **Euclidean**. Euclid stated that a line is the shortest distance between two points. Euclidean distance is represented in Equation (1) [22].
  (1) $$Euclidean=\sqrt{\sum_{i=1}^{n}|P_{i}-Q_{i}{|}^{2}}$$
  where $P_{i}$ and $Q_{i}$ are components of an Euclidean vector indexed with *i*; and *n* is the sample size.
- **Pearson**. It is a measure of the strength of a linear association between two variables. In a broad sense, the Pearson correlation coefficient returns the distance of all data points that best fit through data. Its representation is given by Equation (2)  [23].
  (2) $$Pearson=\frac{1}{n-1}\sum_{i=1}^{n}\left(\frac{x_{i}-\bar{x}}{s_{x}}\right)\left(\frac{y_{i}-\bar{y}}{s_{y}}\right)$$
  where $x_{i}$ and $y_{i}$ are single samples indexed with *i*; *n* is the sample size; $\bar{x}$ and $\bar{y}$ are the sample mean of *x* and *y*, respectively; and $S_{x}$ and $S_{y}$ are the sample standard deviation of *x* and *y*, respectively.
- **Cosine**. It is also called the angular metric. It measures the angle between two vectors, i.e., it is the normalized inner product. The cosine similarity metric is represented in Equation  (3) [22].
  (3) $$Cosine=\frac{\sum_{i=1}^{n}P_{i}Q_{i}}{\sqrt{\sum_{i=1}^{n}P_{i}^{2}}\sqrt{\sum_{i=1}^{n}Q_{i}^{2}}}$$
  where $P_{i}$ and $Q_{i}$ are components of a vector indexed with *i*; and *n* is the sample size.
- **Manhattan**. It is also known as rectilinear distance and taxicab norm. It calculates several projections in the mathematical space, where the size of blocks does not affect the distances. The Manhattan distance is represented in Equation (4) [22,24].
  (4) $$Manhattan=\sum_{i=1}^{n}|P_{i}-Q_{i}|$$
  where $P_{i}$ and $Q_{i}$ are components of a vector indexed with *i*; and *n* is the sample size.
- **Chebyshev**. It is also called the chessboard distance in 2-D or minimax approximation. It was derived by Pafnuty Lvovich Chebyshev. This distance is used when the value of *P* tends to infinity. Its representation is given by Equation (5) [22].
  (5) $$Chebyshev=max_{i}|P_{i}-Q_{i}|$$
  where $P_{i}$ and $Q_{i}$ are components of a vector indexed with *i*.

### 2.3. POI (Point-of-Interest) and Smart POI (Smart Point of Interaction)

The concepts about POI, Smart POI, and Smart Spot will be introduced. Similarly, a brief comparison between a POI and a Smart POI will be presented.

As mentioned above, the recommendation algorithm will deal with Google’s Physical Web technology characteristics [1]. Such technology is currently implemented in the device that will be deployed in Ceutí with the aim of turning Ceutí into a smart tourist destination [25]. This device is called Smart Spot [9].

A Smart Spot is a device that sends push notifications with digital content, like a URL (Uniform Resource Locator), through Bluetooth and Wi-Fi signals to a smartphone—without the need to install native Apps—to generate a physical space of information for users around it; i.e., an interactive area called Smart POI [26]. These solutions aim that visitors and citizens can interact with physical entities through their smartphones, and therefore, can improve their user experiences, for instance, in the tourism sector [9].

**Figure 1:**
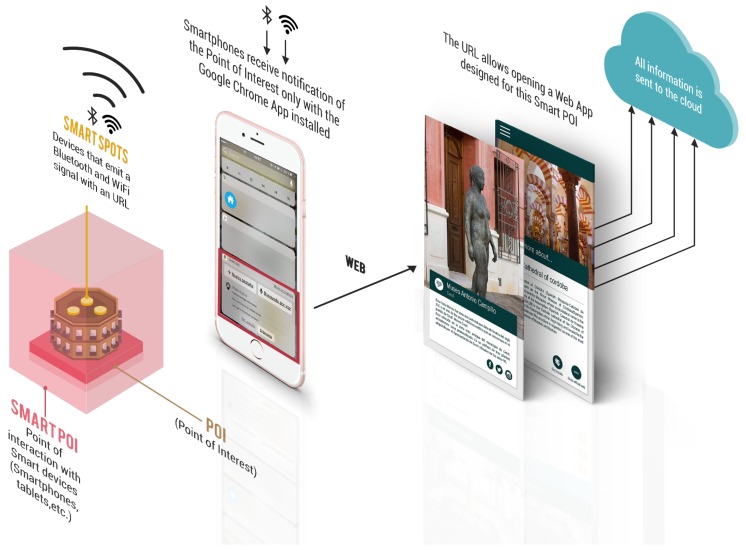
Schema of the relationship among Smart POI, Smart Spot, and POI.

Thereby, a Smart POI is established as a smart interaction area between the users (citizens and visitors) and a specific physical point, identified by a Smart Spot [8,9], while a POI is defined as an interesting place for the user [2]. Hence, a Smart POI allows generating a multidirectional communication channel among citizens, visitors, and city managers as well as boosting open tools for co-creation and culture dissemination [26]. Then, by placing a Smart Spot on a POI, this POI will become a Smart POI, being these Smart POIs (famous landmarks from Ceutí) the objective of this study. In Figure 1, a graphic representation of the relationship among these concepts for this approach is shown.

In addition, to show the field of application of Smart Spots, some examples of entities that can become a Smart POI in two scenarios are given. For tourism sector, POIs such as restaurants, tourist spots, stores, and movie theaters [2,6] can be Smart POIs. For smart cities, entities such as traffic lights, street lights, and another urban infrastructure can become a Smart POI, although they cannot be a possible POI for a citizen or visitor, they can be relevant for city managers. These references are a clear example of how a Smart Spot enriches an entity that can be of interest to a citizen, visitor, or city manager according to a certain context [26]. Consequently, an analysis of the entities that will be considered as Smart POIs in a certain field of application has to be done beforehand.

In summary, the three major advantages of incorporating Smart POIs into different contexts are listed below:

1. Any entity can be a Smart POI when placing it on a Smart Spot.
2. A Smart POI provides a smart interaction area between entities and people through a smartphone.
3. A Smart POI can represent both an interesting place for a visitor and an urban infrastructure relevant for a city manager according to a certain context.

## 3. The HyRA’s Approach

The proposal and development of HyRA are described below.

The main goals of this work are to propose and to develop an algorithm that recommends a Smart POI list for a user according to the user preferences, the Smart POIs’ contextual information such as categories and geographical information, and the characteristics of Smart Spot in conjunction with the definition of Smart POI. Hence, the hypothesis of this proposal is the following: the adaptation of a user-based CF algorithm integrating an average aggregation operator constituted of different similarity and distance measures allows dealing with the user preferences as well as with the characteristics of the technology used in this work, and the incorporation of the Smart POIs’ categories and the geographical influence factor into the modified user-based CF algorithm allows addressing the Smart POIs’ contextual information.

Therefore, on the one hand, the proposal of the HyRA’s approach is based on both the concept of Smart POI and the Smart Spot device. On the other hand, it is established that user preferences are obtained through ratings given by a user to Smart POIs in Ceutí due to the traditional recommendation algorithm chosen to address them. In addition, the similarity and distance measures that will be used in the average aggregation operator have also been defined. These measures are Euclidean distance, Cosine similarity, Spearman correlation, Pearson correlation, Manhattan distance, Bray–Curtis distance, Canberra metric, Chebyshev distance, and Squared Euclidean distance.

### 3.1. User-Based CF: Analysis and Description

The description and pseudocode of HyRA are provided. Similarly, the assumption considered to define the proposed algorithm’s approach is explained.

Initially, the HyRA’s approach was oriented entirely to the POI recommender systems since the Smart POIs defined for this research are POIs of the heritage of Ceutí. However, considering the characteristics of the technology implemented into a Smart Spot, one main assumption was established.

- In the POI recommendation systems, user preferences are reflected and inferred by the frequency of check-in at locations [2,6]. For this scenario, such preferences can be obtained from Smart Spot through interaction between it and the user’s smartphone. Nevertheless, since Smart Spot constantly emits signals to the mobile devices of the users [9], the user’s smartphone can receive all the signals that any Smart Spot emits. Therefore, the interaction between a user and a Smart POI (check-in) does not necessarily indicate interest on that Smart POI, but only that the user is close to it. Consequently, to get the user preferences using Smart Spot, a solution based on the traditional recommendation systems approach was proposed. This is, to have explicitly the ratings for the items [2] considering to Smart POIs as items.

Accordingly, a traditional recommendation approach was defined for dealing with the user’s explicit preferences. As a result, an approach based on the user-based CF algorithm was chosen and adapted because it is the one most used by researchers to address the recommendation based on ratings [2]. To this end, on the one hand, the five-star rating system was incorporated into the surveys since it is the online explicit feedback mechanism that allows collecting more feedback from users [27]. On the other hand, the implementation of the user-based CF algorithm was based on the development reported by Caraciolo [28], using the NumPy package [29], the SciPy library [30], and the Scikit-learn library [31]. The description and pseudocode of HyRA are given below.

Firstly, the user-based CF algorithm is described. Secondly, the user-based CF algorithm with the average aggregation operator is described. Then, the user-based CF algorithm with the average aggregation operator complemented with Smart POIs’ categories is introduced. Lastly, the HyRA’s approach, the user-based CF algorithm with the average aggregation operator complemented with Smart POIs’ categories and with geographical influence, is presented.

1. Data gathering process. The datasets that serve as input for HyRA are loaded: the Smart POIs located in Ceutí and the users’ ratings for each Smart POI. Subsequently, a ground-truth subset was built from the dataset that concentrates all users’ ratings for each Smart POI. There are randomly extracted from one to 11 rated Smart POIs from each user to compose the ground-truth subset, where only Smart POIs whose rating values oscillate between three to five are conserved. This with the aim of leaving behind Smart POIs that are not of interest for a user and that are represented with a ranked below three. By setting to 11 the maximum number of Smart POIs that can be extracted, a total maximum of 70% of the rated Smart POIs of each user is retained to represent their preferences. This ground-truth subset is taken as if the user had only rated this number of Smart POIs. The remaining Smart POIs of each user are used as not visited (not rated) Smart POIs that can be recommended by HyRA. In addition, these remaining Smart POIs and their rated values were preserved in a separate subset to compare the true rated Smart POIs against the Smart POIs recommended by HyRA. Algorithm 1 provides the pseudocode of this phase.
2. Compute similarities between users. A comparison between a user with the rest of them is performed to obtain the *N* users who have most similar preferences with him/her. The rationale behind this is that users who have similar values to a certain user share similar preferences [2]. Thus, it is more likely that the Smart POIs recommended by these similar users matches the preferences of the specified user. To find those users that share analogous preferences with a specific user, a paired comparison of their ratings of Smart POIs is carried out. This comparison iterates through each available user in the dataset to retain all Smart POIs that are presented in the preferences of both users. Then, the ratings of the two users’ Smart POIs are compared by using one distance or similarity measure. Independent experiments are carried out using the following measures: Euclidean distance, Pearson correlation, Cosine similarity, Manhattan distance, and Chebyshev distance. After the paired comparison, a descending list of similarity values among users is obtained per each distance or similarity measure. The similarity values closer to 1 indicate that both users share more preferences in common, while similarity values closer to 0 express the opposite. Algorithm 2 provides the pseudocode of this process.
3. Recommend Smart POIs. For each available user in the dataset—excluding the user that is selected for giving recommendations—are extracted the Smart POIs that the selected user has not visited. Then, each Smart POI not visited is ranked through a weighted mean. The weighted mean contemplates the rating of Smart POI and the similarity value of the user that has been compared to the selected user. Consequently, a descending list of *N* ranked Smart POIs is obtained. From this list, the Top-5 Smart POIs are recommended to the specific user. As a result, only the Smart POIs that could be interesting for the specific user are recommended.

**Algorithm 1** HyRA—Data gathering process.groundTruthSubset←{}notVisitedSmartPOIs←{}**function** LoadDatasets    T←CeutiPOIsDataset    P←UsersRankedPOIsOfCeutiDataset    categories←CategoriesOfEachPOIofCeuti    locations←LocationsOfEachPOIofCeuti    M←T⋂P    **for** user in *P* **do**        userSelectedPOIs←RandomPOIs(M)        groundTruthSubset←groundTruthSubset⋃{(userSelectedPOIs)}        notVisitedSmartPOIs←notVisitedSmartPOIs⋃{(P−userSelectedPOIs)}    **end for**    **return** groundTruthSubset,notVisitedSmartPOIs,categories,locations**end function**

**Algorithm 2** HyRA—Compute similarities between users.
descendingListOfSimilarUsers←{}similarityDistances←{Pearson,Euclidean,Cosine,Manhattan,Chebyshev}**function** GetSimilarUsers(specificUser,otherUsers,groundTruthSubset,similarityMetrics)    **for** distance in similarityDistances **do**        listOfSimilarUsers←{}        **for** user in otherUsers **do**           sharedPOIs←GetCommonPOIs(specificUser,user,groundTruthSubset)           userSimilarityValue←CalculateSimilarityMetric(specificUser,user,sharedPOIs,           distance)           listOfSimilarUsers←listOfSimilarUsers⋃{(user,userSimilarityValue)}        **end for**        descendingList←DescendingSort(listOfSimilarUsers)        descendingListOfSimilarUsers←descendingListOfSimilarUsers⋃{descendingList}    **end for**    **return** descendingListOfSimilarUsers**end function**

### 3.2. User-Based CF with the Average Aggregation Operator

1. Data gathering process. Same process as described in Section 3.1 and presented in Algorithm 1.
2. Compute similarities between users. Same process as described in Section 3.1 and presented in Algorithm 2.
3. Recommend Smart POIs. For each available user in the dataset excluding the user that is selected for giving recommendations, the Smart POIs that the selected user has not visited are extracted. Then, each Smart POI not visited is ranked through a weighted mean. The weighted mean contemplates the rating of Smart POI and the similarity value of the user that has been compared to the selected user. Consequently, a descending list of *N* ranked Smart POIs is obtained. This process is carried out for all similarity and distance measures previously described in Section 2.2 as well as for the Spearman correlation, Bray–Curtis distance, Canberra metric, and Squared Euclidean distance. Thus, nine descending lists of *N* ranked Smart POIs are computed. Afterwards, the frequency of appearance of all Smart POIs embraced in these descending lists is calculated with the objective that all frequencies of the Smart POIs are averaged by the total number of measures used. Lastly, the Top-5 Smart POIs from the final descending list are recommended to the specific user.

### 3.3. User-Based CF with the Average Aggregation Operator + Smart POIs’ Categories

1. Data gathering process. Same process as described in Section 3.1 and presented in Algorithm 1. Furthermore, the Smart POIs’ categories dataset is loaded. Such a dataset is described in Section 6.3.
2. Compute similarities between users. Same process as described in Section 3.1 and presented in Algorithm 2.
3. Recommend Smart POIs. Firstly, all categories of the Smart POIs visited by the user selected to give recommendations are extracted. Then, the Smart POIs’ categories are ranked according to their frequency of appearance. Thus, a descending frequency list of the Smart POIs’ categories is obtained. Thereupon, for each similarity or distance measure, a list of similar users is obtained, who best resemble the specified user according to the procedure described in the previous step. Afterwards, the categories of each Smart POI present in the preferences of each similar user are ranked according to the descending frequency list of the Smart POIs’ categories of the specified user. Finally, all Smart POIs of each similar user are sorted to obtain those that better resemble the specified user preferences. That is, Smart POIs whose categories are closer to the rated Smart POIs’ categories of the specified user are more likely to be recommended. Consequently, a descending list of *N* Smart POIs ranked by their categories is obtained. From this list, the Top-5 Smart POIs are recommended to the specific user.

### 3.4. HyRA

1. Data gathering process. Same process as described in Section 3.3 and presented in Algorithm 1. Furthermore, the Smart POIs’ geographical location dataset is loaded. Such a dataset is described in Section 6.1.
2. Address geographical influence. All Smart POIs loaded in the data gathering process are clustered using K-means with the Euclidean distance. Due to the geographical distribution of Smart POIs, only three clusters are enough to embrace them all. The calculation of the optimal number of clusters is beyond the scope of this paper. As a result, a list containing the cluster number to which each Smart POI belongs is obtained. Then, the cluster’ number of each Smart POI visited by the specific user is extracted. Subsequently, the clusters’ numbers visited by the chosen user are compared against the clusters’ numbers of the Smart POIs of the rest of users. As a result, the users that share at least *N* Smart POIs visited in common with the specific user are retained. Here, it is important to mention that the value of *N* is calculated as follows: one plus the result of the number of clusters visited by the specific user divided by two. This metric has two purposes: first, to ensure that the Smart POIs geographically closer to users location preferences are retained for a possible recommendation; and, second, to decrease the computational calculations that the recommendation algorithm has to perform. Consequently, a list of users that have visited Smart POIs geographically closer to the Smart POIs of a given user is obtained.
3. Recommend Smart POIs. Finally, the procedure described in Section 3.3 is performed to obtain the Top-5 Smart POIs that are going to be recommended, except step 1. As a result, a Smart POI list that could be interesting for the specific user is recommended. Algorithm 3 provides the pseudocode of this process.

**Algorithm 3** HyRA—Recommend Smart POIs.clusterDistances←{Euclidean}**function** Recommend(specificUser,otherUsers,groundTruthSubset,similarityMetrics,categories,)    locations,descendingListOfSimilarUsers)    clusters←GetClusters(locations,clusterDistances)    clusterPOIsVisitedBySpecificUser←GetVisitedClusters(groundTruthSubset[specificUser],    clusters)    mustSharedPOIs←(numberOfClustersVisitedBySpecificUser/2)+1    possibleSimilarUsers←{}    **for** user in otherUsers **do**        clustersOfPOIsVisitedByOtherUsers←GetVisitedClusters(groundTruthSubset[user],        clusters)        sharedPOIs←ComparePOIs(clusterPOIsVisitedBySpecificUser,        clustersOfPOIsVisitedByOtherUsers)        **if** sharedPOIs>=mustSharedPois **then**           possibleSimilarUsers←possibleSimilarUsers⋃{user}        **end if**    **end for**    rankedPOIsCategoriesOfSpecificUser←FrequencyRankCategoriesOfSpecificUserPOIs(    specificUser[categories])    descendingListOfSimilarUsers←GetSimilarUsers{specificUser,otherUsers,    groundTruthSubset,similarityMetrics}    **for** user in descendingListOfSimilarUsers **do**        **for** poi in user **do**           categoriesOfPOI←GetCategoryOfPOI(poi[user])           poiRankedCategorie←RankCategorie(rankedPOIsCategoriesOfSpecificUser,)           categoriesOfPOI)           listOfPOIs←listOfPOIs⋃{poiRankedCategorie}        **end for**    **end for**    descendingListOfPOI←SortDescending(listOfPOIs)    recommendationResults←Top5(descendingListOfPOI)    **return** recommendationResults**end function**


## 4. Experimental Scenario Based on Surveys

A background about figures and data defined for conducting the test phase of HyRA is introduced. Later, the experiments defined for assessing this proposal are described.

### 4.1. Project Background

This section aims to present the project’s main background since the methodology proposed and used to study the application scenario in Ceutí was addressed and discussed in [11]. Such work describes the selection of POIs in Ceutí, the definition of the target audience as well as the sampling methods for this scenario, and the design of the survey. Therefore, only brief statements and key figures to introduce the experimental scenario are provided below.

- Selection of POIs. 16 POIs in Ceutí were defined as Smart POIs. Information about these Smart POIs is presented in Section 6.1.
- Definition of the target audience. Two types of tourists were included in the total target audience: Residents in Spain (86.4%) and Non-residents in Spain (13.6%). On the one hand, the resident target audience was the population of the Región de Murcia ≥18 years old. On the other hand, the non-resident target audience was defined as non-resident travelers in Spain.
- Definition of representative sampling (surveys). The conditions to ensure the building of a database representative of the target audience were defined as follows:
  - The non-probabilistic and cluster-based sampling methods were selected to conduct the surveys. This decision was based on the target audience is hard to identify and the sample is a pilot study [32].
  - The 6.75% margin of error was defined to ensure a representative sample of the target audience. Therefore, the number of surveys to be collected was estimated at 200, of which 173 people must be resident travelers in Spain (86.4%) and 27 people must be non-resident travelers in Spain (13.6%).
  - The 27 surveys for non-resident travelers in Spain were collected globally while the 173 surveys for resident travelers in Spain were divided into clusters. That is, three clusters were considered for this scenario, i.e. 18–30, 31–50, and >50, which also were divided into women and men. Hence, the number of surveys per cluster is shown in Table 2.
  - The survey was designed and managed online via Google Forms (https://www.google.com/intl/en/forms/about/), and structured in both Spanish (https://goo.gl/VrC0ve) and English (https://lnkd.in/dzqVyJD) language to facilitate its dissemination.

### 4.2. The Surveys and the HyRA Evaluation Scenario

The experimental scenario will be divided into two phases: survey evaluation and HyRA test.

On the one hand, to know the effectiveness of the surveys, a pilot dissemination phase is considered. In this phase, the respondents will be encouraged to provide an explicit feedback about their appreciation regarding the surveys’ design and subject-matter, since the implicit feedback will be given by their answers. Subsequently, a period of up-to-date of both surveys is proposed for finally disseminating them to the target audience.

On the other hand, with the aim of evaluating the Smart POI recommendations given by HyRA, a scenario constituted of different tests is designed. These tests include the use of diverse distance and similarity measures, the Smart POIs’ categories, and the geographical influence factor. For this purpose, the following steps are proposed.

1. Extraction of a ground-truth subset of ratings on Smart POIs of each user. With the aim of counting on a ground-truth to assess the recommendation algorithm, the Smart POIs dataset is divided into two. The ground-truth subset is obtained by randomly select up to 11 Smart POIs from each user whose rates vary from three to five stars. By doing this, we can capture approximately the 70% from the 16 Smart POIs ratings given by the users. The aim of this subset is to serve as a ground-truth dataset that allows the recommendation algorithm to have a representation of the preferences of each user. The remaining Smart POIs of each user are used as not visited (not rated) Smart POIs that can be recommended by the recommendation algorithm. The original ratings that each user gives to each Smart POI—which belong to this last subset—are preserved to later compare the recommendations provided by the recommendation algorithm.
2. Selection and implementation of a set of similarity and distance measures to provide the Smart POI recommendation. The objective of this activity is to calculate the first recommendations for this scenario. Experiments are carried out by using each similarity and distance measure described in Section 2.2. Furthermore, the following measures were also tested: Spearman correlation, Bray–Curtis distance, Canberra metric, and Squared Euclidean distance. First, the ground-truth subset is obtained as described above. Then, for each user, his/her recommendations are calculated with each similarity and distance measure. The procedure and description of the algorithm is found in Section 3.1.
3. Incorporation of the validated similarity and distance measures into the average aggregation operator. The aim of this activity is to increase the proposed recommendation algorithm precision. For this experiment, all similarity and distance measures described in Section 2.2 are concentrated into an average aggregation operator as described in Section 3.2. In this experimental phase, one hundred executions are performed in order to compare the user-based CF algorithm with the average aggregation operator against its counterpart with one similarity or distance measure at a time. Each execution is independent of the others, that is, each execution calculated its own random ground-truth subset that is used at that time in both versions of the proposed algorithm.
4. Definition and integration of the Smart POIs’ categories to the proposed recommendation algorithm. The aim of this activity is to increase the proposed recommendation algorithm precision. In this test scenario, the Smart POIs’ categories are taken into account and added to the recommendation algorithm supplemented with the average aggregation operator as described in Section 3.3. In addition, one hundred executions are performed in order to compare the proposed recommendation algorithm supplemented with the average aggregation operator against its counterpart that adds Smart POIs’ categories. Each execution is independent of the others, that is, each execution calculated its own random ground-truth subset that is used at that time in both versions of the proposed algorithm.
5. Implementation of the geographical influence factor in the proposed recommendation algorithm. The aim of this activity is to increase the proposed recommendation algorithm precision. In this phase, the Smart POIs’ locations are integrated into the proposed recommendation algorithm that considers the Smart POIs’ categories. The algorithm description can be reviewed in Section 3.4. Consistently, one hundred executions are performed to compare the proposed recommendation algorithm supplemented with both the average aggregation operator and the Smart POIs’ categories against the recommendation algorithm that adds the geographical influence factor (HyRA). In addition to carrying out the same executions, the results of the recommendation algorithm supplemented with the average aggregation operator against the results of HyRA are compared. Each execution is independent of the others, that is, each execution calculated its own random ground-truth subset that is used at that time in the three versions of the proposed algorithm.
6. Compare the different approaches of the recommendation algorithm. To provide the version of the recommendation algorithm that delivers better recommendations to all users, the results of all implementations previously described are compared. The first step is to sort in descending order the Smart POIs preferences of each user contained in the not visited (not rated) dataset, this with the purpose of obtaining the preferences of each user from the highest to the lowest. Subsequently, the original rating that users granted to each Smart POI recommended by each algorithm per each user is extracted. Consequently, a list that concentrates the Smart POI recommendations with the original ratings for each version of the recommendation algorithm is obtained. Thus, the algorithm whose lists of recommendations deliver the Smart POIs with higher ratings for all users stands as the best approach for this study.

## 5. Results and Discussion

The results obtained from the proposed experimental phase as well as an analysis of the same from both the point of view of the user experience and the recommendation algorithm approach are described.

### 5.1. Surveys: Dissemination and Analysis

A pilot dissemination of the survey in Spanish was carried out with 10 residents in Spain and two foreign people to gather feedback about the design and subject-matter, mainly. Once the survey in Spanish was improved, the survey in English was carried out from the final survey in Spanish. Likewise, three foreign people performed an analysis of the subject-matter to ensure the clarity of the questions in this language. During these reviews, several changes were suggested.

- Spanish version
  - “Age” question. In the first surveys, the birthdate was asked to the respondents. However, this field was changed to the four age ranges established (<18, 18–30, 31–50, and >50) to directly do the clustering of each participant.
  - “Residence” question. The type of format to introduce this answer was specified since sometimes, only the city, country, or locality was typed by the respondent, entailing possible issues to determine the residence of the participant.
  - Sort the questions. The questions related to the tourism in the Región de Murcia and Ceutí “Do you usually tour the Region of Murcia (Spain)?”, “Have you ever visited Ceutí?”, and “if you visited Ceutí, what was the reason for the visit?” were realigned. Firstly, these questions were located between the personal information questions and the SNS questions; thus, some respondents asked if the questions related to the tourism in the Región de Murcia as well as Ceutí and the questions about the usage of SNS were associated, due to their answers could change according to this condition. Hence, to clarify that questions corresponding to the usage of SNS were formulated to know the user preferences in general, these three questions were located after the SNS questions.
  - New options for the answers. Two situations were presented: people from Ceutí and people who had never visited Ceutí answered the survey. Therefore, respondents suggested incorporating “I am from Ceutí” for the “have you ever visited Ceutí?” and “what was the reason for the visit?” questions, as well as “I have not visited Ceutí” for the last question. In addition, in the “what social networks do you use to publish your location during your travels or visits?” question was proposed to add the Twitter option. Such suggestions were integrated into the survey.
  - Information about Ceutí. A brief introduction about Ceutí was described in the have you ever visited Ceutí? question to contextualize foreign respondents.
- English version
  - “Residence” question. The type of format was modified to indicate to the user only writing his/her country.
  - Re-formulated question. “Do you usually tour the Region of Murcia (Spain)? was rephrased to have you ever visited the Region of Murcia?”
  - Points of tourist attraction. The names of these points were translated for their identification, although the original name was also maintained.

After incorporating the changes suggested by the pilot target audience, both surveys were disseminated. These surveys were delivered from 6 April 2017 to 21 April 2017 through the following media:

- SNS: HOP Ubiquitous, town council of Ceutí, and Tecnologico de Monterrey.
- Instant messaging (WhatsApp): people involved in the project (HOP Ubiquitous and Tecnologico de Monterrey).
- E-mail: people involved in the project (HOP Ubiquitous, Tecnologico de Monterrey, and town council of Ceutí).

Considering that the target audience should be composed of residents from the Región de Murcia as well as foreign people, some groups were selected to distribute the surveys.

- People related to the Spanish members of the project located in different geographical locations from the Región de Murcia.
- People related to the Mexican members of the project located in Mexico.
- People identified by the town council of Ceutí.
  - Members of transnational meetings of the town council of Ceutí’s European projects.
  - Members of transnational meetings of the European projects in which the Ceutí’s IES is involved.
  - Members of the relations between families with the St Berthevin City in France.

**Figure 2:**
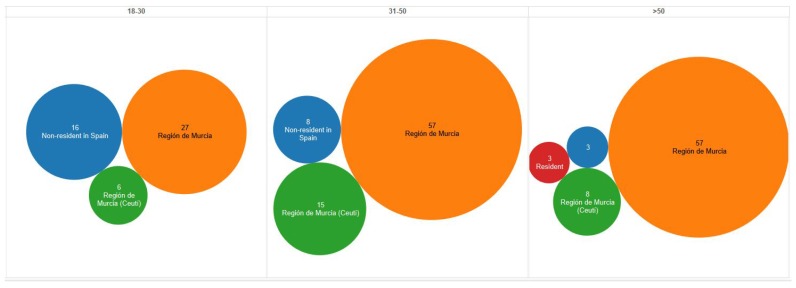
Responders’ residence profile.

The total amount of established surveys (200) was surpassed and the respondents’ locations confirm that the aim of surveying people belonging to the Región de Murcia was, mostly, achieved. Consequently, the study provides a global vision about the target audience preferences, where such respondents can be potential visitors to the town of Ceutí. However, although more than 200 surveys were collected, when building the clusters defined for each age range, the >50 age range clusters could not be completed successfully with only residents from the Región de Murcia. Hence, taking into account that people resident from Spain (not belonging to the Región de Murcia) also participated in the study, the missing user profiles were obtained of this group of respondents. Therefore, three Spanish profiles non-resident in the Región de Murcia were introduced to these clusters. This fact can be appreciated in Figure 2.

To conclude, these surveys, in addition to supporting the building of the dataset related to the target audience preferences, also contributing to one of the capacities that the Internet of Things (IoT) presents to improve any sector: the data collection about the user [33]. As a result, two datasets are provided from this work: one dataset consisting of the preferences of 200 people and one dataset composed of information corresponding to 16 Smart POIs.

### 5.2. HyRA: Analysis and Discussion

To have a varied set of similarity and distance measures with which to search for better recommendations, the following measures are implemented:

- Pearson correlation
- Euclidean distance
- Cosine similarity
- Spearman correlation
- Manhattan distance
- Bray–Curtis distance
- Canberra metric
- Chebyshev distance
- Squared Euclidean distance

Afterwards, a function to divide the set of ratings given by a user has been introduced. That is, two subsets of the global set (16 ratings) are generated, one with 70% of the ratings and another with 30%, 11 ratings and 5 ratings, respectively. The largest set is assigned to the similarity calculation among users while the smallest set will be maintained to make the comparison between the recommendations provided by the algorithm and this set.

However, these parameters were modified because the number of Smart POIs recommended was always the same: five. Then, the only observed change was the similarity value among the same Smart POIs since others Smart POIs could not be included as only five Smart POIs were available to recommend. Hence, a variation in the process of producing these subsets was introduced: the number of ratings to form the subset assigned to the similarity calculation would be random from 1 to 11. In this way, new Smart POI recommendations were ensured.

An example of the results obtained from this analysis is provided below. In Table 3 are presented the ratings given by the responder identified by User-CEUTI-1 for each Smart POI and in Table 4 as well as Table 5 are shown the recommendations suggested for this user. Such recommendations are labeled from 1 if it is the least recommended to 5 if it is the most recommended.

Where:

1. Smart POIs used for the similarity calculation = {Stepping Strong, Allegory of Life, Arabic Ruins of Ceuti, Hermitage of San Roque, My Metaphysical Garden, Queen Mariana, The Canning Woman, Torso}—eight Smart POIs
2. Smart POIs available for the recommendations = {Apothecary’s Noria, “7 Chimneys” Museum, Tribute to the Emigrant, The Mural of San Roque, “Santa Maria Magdalena” Church, Children bathing in La Acequia of Ceuti, “La Conservera” Contemporary Art Museum, “Miguel de Cervantes” Sculpture}—eight Smart POIs

Accordingly, for this example, the Cosine, Manhattan, Bray–Curtis, Canberra, Chebyshev, and Squared Euclidean measures provided the same recommendations.

1. 5—“La Conservera" Contemporary Art Museum
2. 4—”Santa Maria Magdalena” Church
3. 3—”7 Chimneys” Museum
4. 2— Apothecary’s Noria
5. 1—Children bathing in La Acequia of Ceuti

Subsequently, the results obtained by the user-based CF algorithm against the results obtained by the user-based CF with an average aggregation operator are compared to detect the algorithm that provides best Smart POI recommendations. To perform the comparison, both algorithms are executed one hundred times with their independent ground-truth subset as described in Section 4.2.

### 5.3. Smart POI Recommendation through User-Based CF with an Average Aggregation Operator

After performing the experiments described in Section 4.2, the results of all executions of user-based CF and the user-based CF with an average aggregation operator (CF + AO) are compared.

Firstly, experiments are performed with nine similarity and distance measures. Table 6 shows the total counts in which each algorithm wins over the other, the total counts in which the recommendation results end up in a tie, and the counts in which each individual distance wins across all users. Additionally, the count of no comparisons across all users is presented. If it is not possible to calculate any similar user for a given distance, then it is not possible to recommend any Smart POI. Thus, in the absence of recommendations, the comparison of results is not performed.

In Table 6, it can be noticed that the recommendations of Smart POIs made through the user-based CF algorithm best resemble, in general, the preferences of all available users. It is worth mentioning that the Euclidean distance brings results that better resemble the users’ preferences for more than a half of executions of the experiments. Furthermore, and strictly speaking, if it is not possible to perform a comparison due to the lack of Smart POI recommendations through the user-based CF, then the user-based CF + AO algorithm stands as the recommendation algorithm that must be used due to its faculty of always deliver a recommendation to the user. Afterwards, the Mean Squared Error (MSE) for each recommendation given by each similarity and distance measure as well as by the user-based CF + AO are computed. Results are concentrated in Table 7.

Results in Table 7 show that the user-based CF with Euclidean distance has the lowest MSE of all distances, and is even lower than the user-based CF + AO algorithm. In contrast, the Spearman correlation has the highest MSE of all measures. Furthermore, it can be noticed that the inclusion of the Spearman correlation into the user-based CF + AO increases the value of its MSE. Thus, additional experiments are performed to obtain a better combination of the similarity and distance measures for the user-based CF + AO algorithm. The experiments are performed by taking out the similarity metric that has the highest MSE value each one hundred executions of the algorithm. Table 8 concentrates the MSE values of these experiments.

In Table 8, it can be noticed that removing one measure ensures the reduction of the MSE. However, the purpose of the aggregation operator is to provide diversity in the recommendations made by the algorithm. Thus, MSE values from each previous version of the user-based CF + AO algorithm are compared to the MSE values of each similarity or distance measure in the respective experiment. Due to lack of space, the results of the best version of the user-based CF + AO algorithm is presented. The best version of the user-based CF + AO algorithm is obtained by using the following five similarity and distance measures: Euclidean distance, Cosine similarity, Chebyshev distance, Pearson correlation, and Manhattan distance. Table 9 shows the results of one hundred executions of the best combination obtained from such experiments, while Table 10 presents the MSE computed for the same experiments.

Table 9 shows that the winning counts difference between the user-based CF algorithm and the user-based CF + AO algorithm is reduced. Furthermore, Table 10 indicates that the MSE of the user-based CF + AO algorithm is significantly decreased, positioning it before the Manhattan distance. Thus, the decision of keeping those five similarity and distance measures is due to: (1) decrement of the MSE value obtained using the nine similarity and distance measures; (2) retaining more than the half of the available similarity and distance measures; and (3) always delivering a recommendation. For these reasons, the user-based CF + AO algorithm with five similarity and distance measures is selected as the basis of the proposed recommendation algorithm.

### 5.4. Smart POI Recommendation through User-Based CF with an Average Aggregation Operator + Smart POIs’ Categories

The inclusion of categories, tags, or topics is another frequent approach used in the literature in order to improve the recommendations performed by various algorithms. Consequently, the Smart POIs’ categories are included in the algorithm that obtained the best recommendation results from the previous experiment. Then, the experimentation phases described in Section 4.2 are performed. Table 11 summarizes the results obtained in the comparison of the user-based CF + AO algorithm against the user-based CF with an average aggregation operator and the Smart POIs’ categories (CF + AO + C). Moreover, a comparison among the user-based CF + AO + C algorithm with the five selected similarity and distance measures and with only the Euclidean distance is included.

Results show that the addition of categories into the recommendation algorithm improves the general resemble of the users’ preferences. It is noteworthy that these two versions of the recommendation algorithm do not present the lack of results; therefore, it is possible to carry out a comparison. Additionally, another finding is presented by using the five similarity and distance measures to generate new recommendations since the proposed algorithm provides better recommendations than only using the distance with the lowest MSE.

### 5.5. Smart POI Recommendation through Geographical Influence + User-Based CF with an Average Aggregation Operator + the Smart POIs’ Categories (HyRA)

In addition to the Smart POI’s categories, the use of geographical influence is also one approach handled in the literature for improving recommendations. Therefore, the geographical influence factor is added to the user-based CF + AO + C recommendation algorithm as described in Section 3.4. Table 12 shows the results of the comparisons between the user-based CF + AO + C and the GI + user-based CF + AO + C (HyRA). Furthermore, Table 13 shows the results of the comparisons between the user-based CF + AO and HyRA.

Results in Table 12 show that for most of the cases to use the recommendation algorithm with or without the geographical influence is indifferent. However, it can notice that integrating the geographical influence factor resembles slightly better the general users’ preferences than the algorithm that does not include it. Additionally, the results of Table 13 corroborate that the use of the geographical influence factor and the Smart POIs’ categories are favorable for the recommendation results. Even though the Smart POIs encompassed in the dataset are located geographically close one to another, the inclusion of geographical influence can provide Smart POI recommendations that suit better the users’ preferences.

Finally, HyRA is compared with another POI recommendation algorithm in the literature that embraces both user-based CF and geographical influence. In Ye et al. [10], unified collaborative recommendation algorithm (USG) and the user preference/geographical influence based recommendation (UG) algorithm are the two algorithms with the best performances. However, the USG algorithm is not chosen to be compared with HyRA because USG comprises a Friend-based Collaborative Filtering, an approach that is not addressed in this work. Therefore, UG is implemented and compared with HyRA by presenting an approach closer to the HyRA approach. Table 14 shows the comparative results between the UG and HyRA algorithms.

According to the results obtained in Table 14, HyRA resembles better the users’ preferences in the dataset. In addition, it is noteworthy that the inclusion of the Smart POIs’ categories and the integration of an average aggregation operator into a Smart POI recommendation algorithm allow providing better recommendations than approaches that only consider the user-based CF algorithm and the geographical influence factor. Thus, the geographical influence + user-based CF with an average aggregation operator + the Smart POIs’ categories (HyRA) stands so far as the best recommendation algorithm for this research approach by surpassing all the approaches included in these experiments in at least 0.04% and 27.39% of the user-based CF with an average aggregation operator + the Smart POIs’ categories and UG [10] algorithms, respectively.

## 6. Materials and Methods

Firstly, information related to the Smart POIs selected for the experimental phase is shown. Subsequently, the dataset composed of the user’s explicit preferences is introduced. Finally, the dataset constituted of the Smart POIs’ categories is presented. The datasets generated in this research work are freely available for download through a GitHub© repository called HyRA datasets (https://github.com/JoAlvaradoU/HyRA-datasets.git).

### 6.1. Smart POIs Dataset in Ceutí

The proposed recommendation algorithm requires knowing information about the Smart POIs that will be considered to carry out the recommendations. Therefore, a dataset composed of 16 Smart POIs previously defined for this work has been generated. In addition, a geographical representation of all of the Smart POIs is included since this proposal also addresses geographical influence. The structure and records of this dataset as well as the visualization of Smart POIs are presented in Appendix A. A brief description of the information contained in the fields of this dataset is provided below.

- **Smart POI Identifier**. The field that identifies the Smart POI and allows establishing a relationship with the user dataset to extract the ratings assigned by each user to these Smart POIs as well as with the Smart POI’s categories dataset to obtain the tags that describe them.
- **Name**. The Smart POI’s title in both English and Spanish language.
- **Location**. The column that indicates the Smart POI’s coordinate in decimal degrees, whose format is [latitude, longitude].

### 6.2. User Preferences Dataset

Regarding the user explicit feedback, a subset of the information collected through the surveys has been extracted to build the user preferences dataset, which is used by the proposed recommendation algorithm in its traditional part. This dataset is made up of the 16 ratings of 200 people. Because the dataset has 3200 records, only the structure and data of one of the respondents are shown in Appendix B. A brief description of its fields is provided below.

- **User Identifier**. The field that identifies the respondent, solely for purposes of the algorithm because no personal information was collected.
- **Smart POI Identifier**. The key to extracting the information from the Smart POIs dataset.
- **Rating**. The given numerical value by the respondent to the Smart POI according to his/her preferences. This value is within the range from 1 to 5, being 1 not interesting and 5 very interesting.

### 6.3. Smart POIs’ Categories Dataset

Concerning the definition of the Smart POIs’ categories, the All Categories section from the Yahoo! Answers website [12] as well as the description of these places were used to build the categories’ dataset for this scenario. As a result, a dataset composed of 13 different categories was structured, where each Smart POI has three or four of the 13 categories already defined, as shown in Appendix C. Such categories are Sculpture, Outdoors, Human, Mural, Museum, Church, Noria, Building, Architecture, Nature, Art, Square, and Park. A brief description of the dataset fields is presented below.

- **Smart POI Identifier**. The key to extracting the information from the Smart POIs dataset.
- **Category-X**. The fields that indicate the category name.

## 7. Conclusions and Further Work

This research provides the proposal and development of a hybrid recommendation algorithm (HyRA) that uses a novel device and technology called Smart Spot and Physical Web, respectively, for the tourism sector.

HyRA is based on both traditional and POI recommendation approaches as it incorporates the user’s explicit preferences (ratings), the Smart POIs’ categories, the geographical influence factor, as well as the characteristics of Smart Spot and Smart POI when suggesting to the user a new Smart POI list to visit. Specifically, with the aim of dealing with the user’s explicit preferences along with the characteristics of Smart Spot and Smart POI, a modified user-based CF algorithm, which consists of merging an average aggregation operator integrated by five similarity and distance measures as a single measure into the user-based CF algorithm, is proposed and validated. In the same way, to encode the Smart POIs’ categories, a filtering method is incorporated, and to unify the geographical influence factor, the K-means algorithm using an Euclidean distance are assembled.

Additionally, with the aim of carrying out the evaluation of HyRA, one survey in Spanish and another in English were disseminated to collect information related to the general user preferences and profiles as well as the user’s specific preferences on the defined Smart POIs in this scenario. As a result, two datasets have been structured and generated according to the real-world scenario in Ceutí: one dataset constituted of the 16 Smart POIs, and the other composed of the ratings provided at 16 Smart POIs per 200 people. In addition, an experimental dataset consisting of 13 categories was built.

These three datasets are used in the experimental cases defined in this proposal and are published for reuse. The evaluation shows that HyRA overcomes all the recommendation approaches assessed.

As future work, the analysis of all the results obtained from the surveys is considered to design an application for the tourism sector in Ceutí oriented to the target audience’s preferences. In addition, the standardization of both the hierarchy of the categories and the categories for Smart POIs is proposed. Furthermore, the incorporation of the time factor into HyRA is contemplated to consider, for example, the museums’ opening hours before recommending them.

## Figures and Tables

**Table 1:** Comparison of the POI recommendation approaches.

Author	Y	R	CI	GI	SI	C	ACD	IS	SDM	UCF+	PSSP	S
Ye et al. [10]	2011	**✗**	✔	✔	✔	**✗**	**✗**	LBSN	✔	**✗** *	**✗**	LBSN
Zheng et al. [16]	2013	**✗**	✔	**✗**	✔	✔	**✗**	LBSN	✔	**✗**	**✗**	LBSN
Liu et al. [5]	2013	**✗**	✔	✔	**✗**	✔	**✗**	LBSN	**✗**	**✗**	**✗**	LBSN
Liu et al. [17]	2013	**✗**	✔	✔	**✗**	✔	✔	LBSN	**✗**	**✗**	**✗**	LBSN
Yuan et al. [7]	2014	**✗**	✔	✔	**✗**	**✗**	✔	LBSN	**✗**	**✗**	**✗**	LBSN
Liu et al. [6]	2015	**✗**	✔	✔	**✗**	**✗**	**✗**	LBSN	**✗**	**✗**	**✗**	LBSN
Zhang and Wang [18]	2015	**✗**	✔	✔	✔	**✗**	✔	LBSN	✔	**✗**	**✗**	LBSN
Guo et al. [15]	2017	**✗**	✔	✔	**✗**	**✗**	**✗**	LBSN	**✗**	**✗**	**✗**	LBSN
Kang et al. [19]	2006	✔	**✗**	**✗**	**✗**	✔	**✗**	Jeju-do Tourist Association from Republic of Korea and surveys from the Internet	✔	**✗** *	**✗**	Tourism
Ying et al. [20]	2012	**✗**	✔	**✗**	✔	✔	✔	LBSN	✔	**✗**	**✗**	Urban areas
Meehan et al. [3]	2013	**✗**	**✗**	**✗**	**✗**	**✗**	✔	WorldWeatherOnline API, Twitter, and users themselves	**✗**	**✗**	**✗**	Tourism
Yu et al. [21]	2016	✔	✔	✔	**✗**	✔	✔	LBSN	✔	**✗** *	**✗**	Tourism
HyRA	2018	✔	**✗**	✔	**✗**	✔	**✗**	Surveys through the Web (test) and Smart Spot in the real-world	✔	✔	✔	Tourism

**Table 2:** Number of surveys assigned to each cluster.

Cluster	Age Range	Men	% Men	Women	% Women
1	18–30	17	9.8266	16	9.2486
2	31–50	37	21.3873	35	20.2312
3	>50	32	18.4971	36	20.8092
Total		86	49.7110	87	20.2890

**Table 3:** User preferences identified by User-CEUTI-1.

Smart POI Name	Rating
Stepping Strong	4
Allegory of Life	2
“7 Chimneys" Museum	4
"La Conservera” Contemporary Art Museum	4
“Santa Maria Magdalena” Church	4
Arabic Ruins of Ceuti	3
Hermitage of San Roque	5
My Metaphysical Garden	4
Apothecary’s Noria	3
Children bathing in La Acequia of Ceuti	4
The Mural of San Roque	3
Queen Mariana	4
The Canning Woman	4
“Miguel de Cervantes” Sculpture	3
Tribute to the Emigrant	3
Torso	5

**Table 4:** Recommendations given by the algorithm to the responder identified by User-CEUTI-1 (I).

Smart POI	Pearson	Euclidean	Cosine	Spearman	Manhattan
Stepping Strong					
Allegory of Life					
“7 Chimneys” Museum		4	3		3
“La Conservera” Contemporary Art Museum	1	5	5	1	5
“Santa Maria Magdalena” Church	4	2	4	2	4
Arabic Ruins of Ceuti					
Hermitage of San Roque					
My Metaphysical Garden					
Apothecary’s Noria	3	3	2		2
Children bathing in La Acequia of Ceuti	5	1	1	3	1
The Mural of San Roque	2			4	
Queen Mariana					
The Canning Woman					
“Miguel de Cervantes” Sculpture				5	
Tribute to the Emigrant					
Torso					

**Table 5:** Recommendations given by the algorithm to the responder identified by User-CEUTI-1 (II).

Smart POI	Bray–Curtis	Canberra	Chebyshev	Squared Euclidean
Stepping Strong				
Allegory of Life				
“7 Chimneys” Museum	3	3	3	3
“La Conservera” Contemporary Art Museum	5	5	5	5
“Santa Maria Magdalena” Church	4	4	4	4
Arabic Ruins of Ceuti				
Hermitage of San Roque				
My Metaphysical Garden				
Apothecary’s Noria	2	2	2	2
Children bathing in La Acequia of Ceuti	1	1	1	1
The Mural of San Roque				
Queen Mariana				
The Canning Woman				
“Miguel de Cervantes” Sculpture				
Tribute to the Emigrant				
Torso				

**Table 6:** Comparison between the recommendations given by the user-based CF against the user-based CF with an average aggregation operator that integrates nine similarity measures.

	Counts of Winning Comparisons	Percentage of Winning Comparisons
Wins of CF across all executions	72,968	44.00%
Winning distance of CF across all executions	Euclidean distance	NA
	with 68/100 executions
Wins of CF + AO across all executions	55,445	33.43%
Draws across all executions	35,848	21.61%
No comparisons across all executions	1599	0.96%

**Table 7:** MSE of each of the nine similarity and distance measures concentrated in the user-based CF algorithm and of user-based CF with an average aggregation operator that integrates the nine measures.

Similarity and Distance Measures, or Algorithm	MSE
Euclidean distance	0.85
Cosine similarity	1.07
Chebyshev distance	1.37
Pearson correlation	1.54
Manhattan distance	1.62
Bray–Curtis distance	1.63
Canberra metric	1.80
Squared Euclidean distance	1.82
Spearman correlation	14.68
User-based CF + AO	2.16

**Table 8:** MSE of different combinations of the similarity and distance measures for the user-based CF with an average aggregation operator.

Number of Measures	MSE
Nine	2.16
Eight	1.69
Seven	1.67
Six	1.60
Five	1.57
Four	1.60
Three	1.22
Two	1.13

**Table 9:** Comparison between the recommendations given by the user-based CF algorithm against the user-based CF with an average aggregation operator that integrates five similarity and distance measures.

	Counts of Winning Comparisons	Percentage of Winning Comparisons
Wins of CF across all executions	65,636	42.53%
Winning distance of CF across all executions	Euclidean distance	NA
	with 60/100 executions
Wins of CF + AO across all executions	55,159	35.74%
Draws across all executions	31,977	20.72%
No comparisons across all executions	1562	1.01%

**Table 10:** MSE of the nine similarity and distance measures concentrated in the user-based CF algorithm and of user-based CF with an average aggregation operator that integrates five similarity and distance measures.

Similarity and Distance Measures, or Algorithm	MSE
Euclidean distance	0.84
Cosine similarity	1.08
Chebyshev distance	1.38
Pearson correlation	1.55
Manhattan distance	1.62
Bray–Curtis distance	1.64
Canberra metric	1.80
Squared Euclidean distance	1.81
Spearman correlation	14.70
CF + AO	1.57

**Table 11:** Comparison between the recommendations given by the user-based CF algorithm with an average aggregation operator against the user-based CF with an average aggregation operator and the Smart POIs’ categories.

	Counts of Winning Comparisons	Percentage of Winning Comparisons
Wins of CF + AO across all executions	6164	22.06%
Wins of CF + AO + C across all executions	9170	32.82%
Wins of CF + AO + C across all executions (Euclidean)	8849	31.67%
Draws across all executions	3760	13.45%

**Table 12:** Comparison between the user-based CF with an average aggregation operator + the Smart POIs’ categories and HyRA.

	Counts of Winning Comparisons	Percentage of Winning Comparisons
Wins of CF + AO + C across all executions	27	0.14%
Wins of HyRA across all executions	36	0.18%
Draws across all executions	19,630	99.68%

**Table 13:** Comparison between the user-based CF with an average aggregation operator and HyRA.

	Counts of Winning Comparisons	Percentage of Winning Comparisons
Wins of CF + AO across all executions	6129	31.12%
Wins of HyRA across all executions	9653	49.02%
Draws across all executions	3911	19.86%

**Table 14:** Comparison between the UG and HyRA recommendation algorithms.

	Counts of Winning Comparisons	Percentage of Winning Comparisons
Wins of UG [10] across all executions	5707	28.99%
Wins of HyRA across all executions	11,099	56.38%
Draws across all executions	2879	14.63%

**Figure A1:**
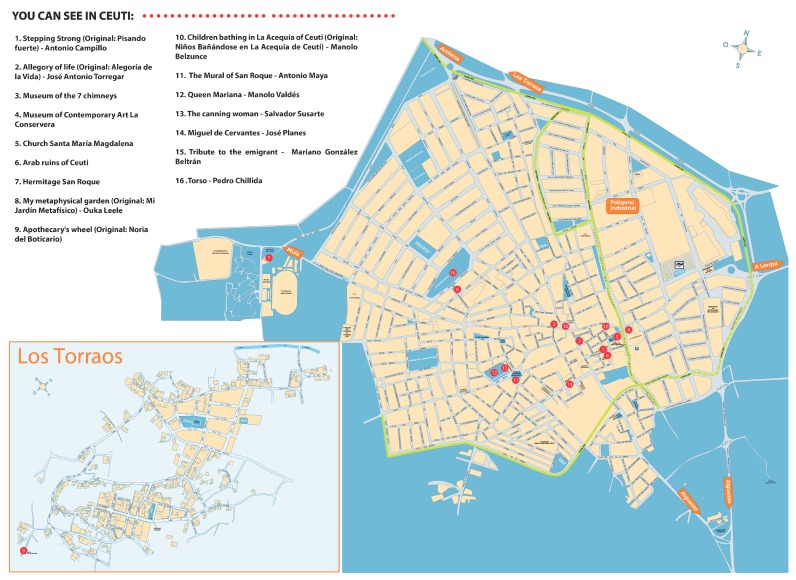
Smart POIs’ geolocation (based on *Map—“7 Chimneys” Museum* [34]).

## Abbreviations

The following abbreviations are used in this manuscript:

CF	Collaborative Filtering
CRTCF	Cross-Region Topic-based Collaborative Filtering
Geo-PFM	Geographical Probabilistic Factor Model
GPS	Global Positioning System
GTAG	Geographical-Temporal influences Aware Graph
GT-BNMF	Geographical-Topical Bayesian Non-negative Matrix Factorization
HyRA	Hybrid Recommendation Algorithm
IoT	Internet of Things
LTSCR	Location and Time aware Social Collaborative Retrieval model
MDPI	Multidisciplinary Digital Publishing Institute
MSE	Mean Squared Error
POI	Point-Of-Interest
Smart POI	Smart Point Of Interaction
SNS	Social Networking Sites
UG	User preference/Geographical influence based recommendation
UPOI-Mine	Urban POI-Mine
URL	Uniform Resource Locator
USG	Unified collaborative recommendation algorithm
VISIT	Virtual Intelligent System for Informing Tourists
WBPR-FD	Weighted Bayesian Personalized Ranking model with visit Frequency and Distance

## Appendix A. Smart POIs Dataset in Ceutí

The structure and records of this dataset are presented in Table A1.

To support the visualization of the Smart POIs in Ceutí, a map with their locations is provided in Figure A1.

**Table A1:** Smart POIs dataset.

Smart POI Identifier	Name	Location
Heritage-ES-Ceuti-1	Stepping Strong	38.078472, −1.270139
	Original: Pisando fuerte	
Heritage-ES-Ceuti-2	Allegory of Life	38.078889, −1.271444
	Original: Alegoría de la Vida	
Heritage-ES-Ceuti-3	“7 Chimneys” Museum	38.079417, −1.272889
	Original: Museo “7 Chimeneas”	
Heritage-ES-Ceuti-4	“La Conservera” Contemporary Art Museum	38.079194, −1.269000
	Original: Museo de Arte Contemporáneo “La Conservera”	
Heritage-ES-Ceuti-5	“Santa Maria Magdalena” Church	38.079056, −1.269528
	Original: Iglesia “Santa María Magdalena”	
Heritage-ES-Ceuti-6	Arabic Ruins of Ceuti	38.078417, −1.27016
	Original: Ruinas Árabes de Ceutí	
Heritage-ES-Ceuti-7	Hermitage of San Roque	38.082111, −1.28466
	Original: Ermita de San Roque	
Heritage-ES-Ceuti-8	My Metaphysical Garden	38.080722, −1.276806
	Original: Mi Jardín Metafísico	
Heritage-ES-Ceuti-9	Apothecary’s Noria	38.100167, −1.287722
	Original: Noria del Boticario	
Heritage-ES-Ceuti-10	Children bathing in La Acequia of Ceuti	38.079389, −1.270056
	Original: Niños Bañándose en La Acequia de Ceutí	
Heritage-ES-Ceuti-11	The Mural of San Roque	38.079833, −1.273306
	Original: El Mural de San Roque	
Heritage-ES-Ceuti-12	Queen Mariana	38.077806, −1.274861
	Original: Reina Mariana	
Heritage-ES-Ceuti-13	The Canning Woman	38.077778, −1.274194
	Original: La Mujer Conservera	
Heritage-ES-Ceuti-14	“Miguel de Cervantes” Sculpture	38.077306, −1.271722
	Original: Escultura “Miguel de Cervantes”	
Heritage-ES-Ceuti-15	Tribute to the Emigrant	38.079472, −1.271917
	Original: Homenaje al Emigrante	
Heritage-ES-Ceuti-16	Torso	38.081444, −1.276944
	Original: Torso	

## Appendix B. User Preferences Dataset

The structure and data of one of the respondents are presented in Table A2.

**Table A2:** User preferences dataset: example of structure for each user.

User Identifier	Smart POI Identifier	Rating
User-CEUTI-1	Heritage-ES-Ceuti-1	4
User-CEUTI-1	Heritage-ES-Ceuti-2	2
User-CEUTI-1	Heritage-ES-Ceuti-3	4
User-CEUTI-1	Heritage-ES-Ceuti-4	4
User-CEUTI-1	Heritage-ES-Ceuti-5	4
User-CEUTI-1	Heritage-ES-Ceuti-6	3
User-CEUTI-1	Heritage-ES-Ceuti-7	5
User-CEUTI-1	Heritage-ES-Ceuti-8	4
User-CEUTI-1	Heritage-ES-Ceuti-9	3
User-CEUTI-1	Heritage-ES-Ceuti-10	4
User-CEUTI-1	Heritage-ES-Ceuti-11	3
User-CEUTI-1	Heritage-ES-Ceuti-12	4
User-CEUTI-1	Heritage-ES-Ceuti-13	4
User-CEUTI-1	Heritage-ES-Ceuti-14	3
User-CEUTI-1	Heritage-ES-Ceuti-15	3
User-CEUTI-1	Heritage-ES-Ceuti-16	5

## Appendix C. Smart POIs’ Categories Dataset

The structure and records of this dataset are presented in Table A3.

**Table A3:** Smart POIs’ categories dataset.

Smart POI Identifier	Category-1	Category-2	Category-3	Category-4
Heritage-ES-Ceuti-1	Sculpture	Outdoors	Human
Heritage-ES-Ceuti-2	Mural	Outdoors	Human	Art
Heritage-ES-Ceuti-3	Museum	Building	Architecture	Art
Heritage-ES-Ceuti-4	Museum	Building	Architecture	Art
Heritage-ES-Ceuti-5	Church	Building	Architecture	Art
Heritage-ES-Ceuti-6	Museum	Building	Architecture	Outdoors
Heritage-ES-Ceuti-7	Church	Building	Architecture	Outdoors
Heritage-ES-Ceuti-8	Mural	Outdoors	Nature	Art
Heritage-ES-Ceuti-9	Noria	Outdoors	Architecture	Nature
Heritage-ES-Ceuti-10	Mural	Outdoors	Human	Art
Heritage-ES-Ceuti-11	Mural	Outdoors	Human	Art
Heritage-ES-Ceuti-12	Sculpture	Outdoors	Human
Heritage-ES-Ceuti-13	Sculpture	Outdoors	Human	Square
Heritage-ES-Ceuti-14	Sculpture	Outdoors	Human
Heritage-ES-Ceuti-15	Sculpture	Outdoors	Human	Square
Heritage-ES-Ceuti-16	Sculpture	Outdoors	Human	Park
